# Impact of tax and subsidy framed messages on high- and lower-sugar beverages sold in vending machines: a randomized crossover trial

**DOI:** 10.1186/s12966-018-0711-3

**Published:** 2018-08-13

**Authors:** Sharna Si Ying Seah, Salome A. Rebello, Bee Choo Tai, Zoey Tay, Eric Andrew Finkelstein, Rob M. van Dam

**Affiliations:** 1 Saw Swee Hock School of Public Health, National University of Singapore and National University Health System, Singapore, Singapore; 2 Department of Medicine, Yong Loo Lin School of Medicine, National University of Singapore and National University Health System, Singapore, Singapore; 3 Investigational Medicine Unit, Yong Loo Lin School of Medicine, National University of Singapore and National University Health System, Singapore, Singapore; 4 Health Services & Systems Research Programme, Duke-NUS Medical School, Singapore, Singapore; 5 NUS Graduate School for Integrative Sciences and Engineering, National University of Singapore, Singapore, Singapore; 6 Department of Nutrition, Harvard T.H. Chan School of Public Health, Boston, MA 02115 USA

**Keywords:** Sugar sweetened beverages, Vending machines, Message framing, Health Behaviours, Crossover trial

## Abstract

**Objective:**

Framing of fiscal incentives has been suggested to be important in influencing purchase decisions. We aimed to examine the effect of framing a modest price difference between high- and lower-sugar beverages as a tax or a subsidy respectively, using messages placed on vending machines to influence beverage purchases.

**Design/setting:**

This is an 11-week randomized crossover trial conducted between August and November 2015, with a two-week run-in period before intervention, targeted at students, staff and faculty of a university campus in Singapore. Twenty-one beverage vending machines were used to implement the intervention involving ‘tax message’, ‘subsidy message’ and ‘no message (control)’. The former two messages suggest ‘a tax for high sugar beverages’ or ‘a subsidy for lower sugar beverages’ respectively. Prices of the beverages offered were fixed at baseline and remained the same in all three experimental conditions: lower-sugar beverage options were priced ~ 10% lower than the corresponding high-sugar option. The machines were randomized to one of the 6 sequences of intervention. Each message intervention period was 3 weeks. The effect of messages was assessed by comparing average weekly units of beverages sold between interventions using mixed effects model.

**Results:**

The average weekly units of high and lower-sugar beverages sold per vending machine were 115 and 98 respectively in the control condition. The percentage of high-sugar beverages sold was 54% in the control, 53% in the tax, and 54% in the subsidy message condition. There was no difference in the weekly units of high-sugar beverages sold for the tax message (− 2, 95% CI -8 to 5, *p* = 0.61) or the subsidy message (0, 95% CI -10 to 10, *p* = 0.96) conditions as compared with the control condition. Similarly, there was no difference in the weekly units of lower-sugar beverages sold for the tax message (4, 95% CI -4 to 13, *p* = 0.32) or the subsidy message (7, 95% CI -4 to 18, *p* = 0.18) conditions as compared with the control condition.

**Conclusions:**

The use of tax and subsidy messages to highlight modest price differences did not substantially reduce high-sugar beverage sales in vending machines on an Asian university campus.

**Electronic supplementary material:**

The online version of this article (10.1186/s12966-018-0711-3) contains supplementary material, which is available to authorized users.

## Introduction

In many populations sugar-sweetened beverages (SSB) are a major source of added sugar that may contribute to excess energy intake in adults [1, 2]. SSBs are defined as beverages that are sweetened with caloric sweeteners which includes regular carbonated beverages, calorically sweetened waters, ready-to-drink coffees/teas, isotonic or sports beverages, energy drinks, and less than 100% fruit juices and drinks [3, 4]. Increased consumption of SSB has been associated with greater weight gain and a higher risk of type 2 diabetes and cardiovascular diseases [5–7]. In 2013, the prevalence of overweight and obese Singaporean adults were 34.3% and 8.6% respectively [8]. Currently, the average daily added sugar intake is 12 teaspoons [9] while the national recommended daily intake of added sugar is eight to 11 teaspoons (40 to 55 g) [10]. One in six Singaporeans consume two or more SSB daily [11] and beverages contribute 60% of Singaporeans’ total added sugar intake [12]. Hence, like many other countries [7] SSB consumption has been a key dietary component targeted in health promotion interventions in Singapore [13]. The Singapore Government has initiated several programs like the Whole-of-Government Healthier Drinks Policy (where lower-sugar beverages are made the default choices in government premises) and the Healthier Choice Symbol (HCS) (voluntary front-of-pack labelling) to promote and facilitate choice of healthier beverages [14]. To qualify for the HCS endorsement, beverages are required to meet the different subcategories’ per 100 g of product sugar content criteria [15].

Different intervention strategies have been employed to nudge populations to select healthier food and beverage options [16, 17]. Nudging is defined as ‘any aspect of choice architecture that modifies individuals’ behaviour without options restriction or significant economic incentives alteration’ [18]. Nudging can be categorized into two types; priming (e.g. increasing healthier products’ visibility, accessibility, availability or a combination of two or all three strategies) and salience (e.g. use of verbal prompts and front-of-pack calorie content labels, traffic light labels or descriptive labels) [19]. The effect of the two types of nudges on food and beverage choices was found to increase healthier food and beverage choices when combined but inconclusive when applied individually from a systematic review which excluded interventions using economic incentives [19]. A recent study suggested that front-of-pack SSB labels (text warning, graphic with text warning, sugar information and Health Star Rating (HSR)) might be useful in reducing young Australian adults’ selection of SSB compared to control (without label) [20]. Though the effect of an HSR label on consumers’ food and beverage choices was found to be weak in an experimental online scenario [21] and real-world setting studies [22, 23]. The finding that the graphic warning labels might be more effective in changing beverage selection compared to other labels is in line with other online choice scenario studies [24–26]. However, implementation of such warning labels in the real-world setting might be challenging due to strong opposition from the SSB and sugar industries [27, 28]. While labelling (using high sugar symbol, text warning label and HSR) had modest effects on SSB purchases in Canadian adults in an experimental marketplace study, increasing prices of SSB via tax (10%, 20%, 30% and variable tax proportional to free sugar level) was found to be associated with a significant reduction in purchase [21].

Price is an important determinant of food and beverages choices [29, 30] that can overwrite considerations regarding nutritional quality [31]. Economic interventions like healthier food/beverage subsidies and unhealthier food/beverage taxation have therefore been promoted as strategies to shift population dietary behaviours to healthier ones [32–34]. Some empirical studies suggest that subsidies increase consumption of healthier foods and taxation decrease consumption of unhealthier foods [34–38]. Tax can be administered in different forms which have different impact on sales; transparent taxes (displayed on the product or shelf e.g. excise taxes and value-added tax) were found to be more efficient in influencing purchase decisions compared to hidden taxes (presented only on bills e.g. sales taxes) in previous studies [39, 40]. In modelling studies, a 5.8% to 15% sales reduction was predicted from 10% SSB taxation [34, 41–44]. Most studies examined the effect of tax and subsidy on SSB consumption in demand system models or online simulations [45]. Demand system models-based studies are based on survey results and simulation studies which uses hypothetical purchasing scenarios might not be reflective of real world situations [34]. Thirty three countries have enacted an SSB tax by 2018, some of which evaluated the results of the taxes implemented [46]. An average *ad valorem* soda sales tax of 10% was associated with a reduction in SSB demand by 6–9% in the first two years of implementation in Mexico [47, 48]. Similarly, a 9% decrease in purchase was also observed from SSB sales data in Berkley, California after SSB were taxed at USD$0.01 per ounce [49]. After an excise tax of USD$0.015 per ounce to the cost of beverages was implemented for two months in Philadelphia, intakes of energy drinks and regular soda were observed to decrease significantly in daily drinkers while daily bottled water drinkers were found to increase in proportion [50]. Economic matters are not uniformly decided on in a logically consistent manner due to existing biases and heuristics in individuals’ perceptions [51]. Opinions or choices can be swayed when topics are portrayed from certain angles to highlight specific features, known as ‘framing effect’ [39, 51–53]. Message framing is a nudging strategy used in behavioural economics and psychological interventions [54]. Few experimental studies evaluated the impact of tax and subsidy messages on SSB sold in real world situations where individuals are exposed to varying message frames [55].

Hence, this study aims to explore the following research question: Will framing a small price difference between high- and lower-sugar beverages as a ‘tax for high sugar beverages’ or ‘subsidy for lower sugar beverages’ increase its salience to consumers and influence their beverage purchase decisions? We conducted this study using beverage vending machines in a university campus setting in Singapore. We hypothesized that the units of high-sugar beverages sold will decrease and the units of lower-sugar beverages sold will increase during both the tax and subsidy message conditions.

## Materials and methods

### Study sample

We included 21 vending machines located within the National University of Singapore campus in our trial. These vending machines were operated by a single vendor, one of the three vending machine owners on campus, and were selected based on their ability to track sales electronically. All machines were situated in areas easily accessible by students, staff and faculty members. The population on this campus is aged 18 years and above and of multiple ethnic backgrounds. Four machines were located at sports facilities, two at canteens, and the rest near lecture halls, laboratories or classrooms. All machines had 30 beverage display slots except for three machines that had 36 slots. The study was conducted in collaboration with the university’s Office of Campus Amenities. We were not required to apply for approval from the Institutional Review Board as we did not include any research participants. Besides, no private, medical, or health information was collected, and there was no more than minimal risk to consumers of the machines and their rights and welfare were not restricted.

### Intervention

**Fig. 1:**
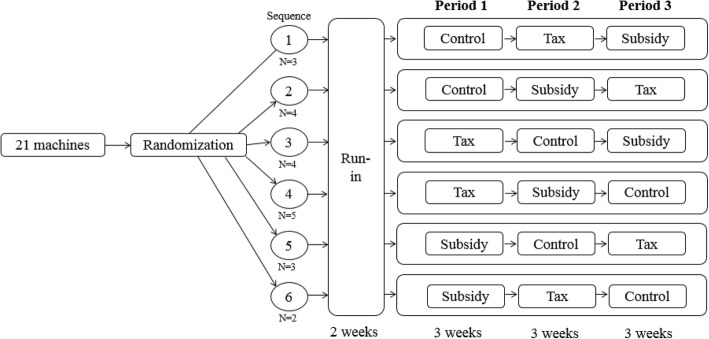
Crossover design of message intervention with six permutations

The study was a crossover trial held over nine weeks (three intervention periods of three-week duration) preceded by the 2-week run-in. The vending machines were randomly assigned to one of six intervention sequences via random permuted blocks of size three with equal allocation for the three interventions (Fig. 1 and Additional file 1: Table S1) by a statistician who was not involved in other aspects of the study conduct. The number of vending machines ranged from 2 machines for sequence 6 to 5 machines for sequence 4. Three conditions were compared: ‘control’, ‘tax’ and ‘subsidy’ messages. Each vending machine was subjected to all three conditions successively according to the sequence allocated. Our research team changed the intervention materials according to the message conditions without revealing the sequences to the vending machine vendors.

Prior to the start of the run-in phase we standardized the types of beverages and the price of the beverages. Specifically, the beverages offered by each machine were standardized to 22 non-alcoholic choices (Additional file 2: Tables S2 and S3); 13 high- and nine lower-sugar beverages. For this study, lower-sugar beverages were defined as drinks with a sugar content of 5 g sugar per 100 ml or less (except for ‘Red Bull Less Sugar’, which contained 12.3 g sugar/100 ml as compared with ‘Redbull Regular’ which contains 16 g/100 ml). Lower-sugar options included purified water, diet soft drinks, zero sugar herbal and green teas, and lower-sugar coffees. Total calories per unit of lower-sugar beverages ranged from 0 to 125 kcal (Additional file 2: Table S3). High-sugar drinks were defined as drinks with a sugar content above 5 g per 100 ml and included sugar-sweetened soft drinks, sports drinks, regular ice teas, energy drinks and sugar-sweetened coffees. The total calories per unit of high-sugar beverages ranged from 69 to 245 kcal (Additional file 2: Table S2). The prices of beverages were also standardized for beverages with counterparts (teas, carbonated drinks, energy drink and coffees): the price of lower-sugar beverages options was SGD$0.10 lower than the corresponding high-sugar option which was typically SGD$1.00. Prior to our study, lower-sugar beverages were priced SGD$0.10 higher than high-sugar beverages. Purified water, priced at SGD$0.70, was the cheapest option in the vending machines. The proportions of lower-sugar beverages in the vending machines was 44% of beverages offered. Throughout the duration of the study, no changes were made to the beverage prices, types of beverages offered, position of beverages in the vending machines, or locations of the machines.

No banners, posters nor stickers were displayed during the control condition (Table 1). During the message conditions, machines had white banners, posters, and bright yellow stickers to highlight the ‘tax’ or ‘subsidy’ in place. Images of the vending machines during different message conditions are shown in Additional file 3: Figure S1. Environmental audits were conducted weekly to check if the correct messages were displayed as per protocol and monitor the state of our vending machines and changes in other competitor machines nearby during intervention. Pre-and post-intervention weekly sales data of each beverage were collected from each machine between end August and November 2015.

**Table 1:** Message elements during control, tax and subsidy conditions

Condition	Banner message^a^	Poster message^b^	Stickers message^c^
Control	No banner	No poster	No stickers
Tax	‘HIGH SUGAR DRINKS ARE TAXED.’	‘THESE HIGH SUGAR DRINKS ARE TAXED!’	‘Cost More’ stickers placed on high-sugar beverages on the selection panels
Subsidy	‘LOWER SUGAR DRINKS ARE SUBSIDISED.’	‘THESE LOWER SUGAR DRINKS ARE SUBSIDISED!’	‘Cost Less’ stickers placed on lower-sugar beverages on the selection panels

^a^Banners (23 × 4 in.) were in white with red font, placed above the top selection panel in machines and referred consumers to poster for the beverages that were ‘taxed’ or ‘subsidised’

^b^Posters (16.5 × 12 in.) were placed at the bottom left corner of the selection panel to highlight the selected beverages that were ‘taxed’ or ‘subsidised’

^c^Stickers (1 × 1 in.) were bright yellow with black font, and positioned below the ‘taxed’ or ‘subsidised’ beverage

### Data analysis

**Fig. 2:**
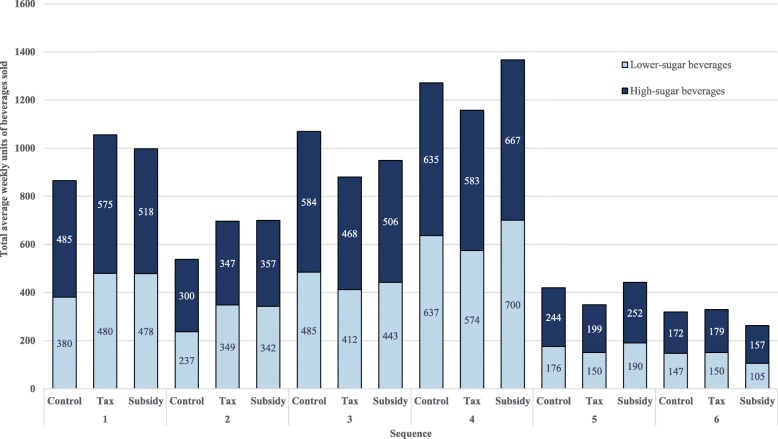
Total average weekly beverage units of high- and lower-sugar beverages sold during different message conditions per intervention sequence

Primary outcome data, weekly units of high- and lower-sugar beverages sold per machine, were converted to average weekly units sold per machine for each message condition prior to analysis. To examine whether messages influenced the sales of beverages, mixed effects model assuming random intercept was conducted for high- and lower-sugar beverages separately. The average weekly units of beverages sold was the dependent variable, the intervention (i.e. control, tax and subsidy message conditions) was regarded as fixed effects with the control as the reference group and variance within each machine contributing to the random effects. Adjustment was made to account for the period effect as there were fluctuations in overall beverage sales (Fig. 2) which affects all machines during the study period. Carryover effects, defined as residual effects from the condition prior to the condition of interest, were tested by comparing the sum of beverage units sold during the nine weeks intervention between the six sequences using the one-way Analysis of Variance (ANOVA) test as previously suggested [56]. It can be assumed that there is negligible carryover effect when there is no difference between the six sequences with respect to the total units of beverages sold. A sensitivity analysis was conducted to explore if message carryover affected the outcome by adjusting for the carryover effect in the multilevel mixed effect models. Hypotheses were evaluated by 2-tailed tests and significance level was set at 0.05. All data were analysed using Stata SE, version 13.0 for Windows (Stata Corp., College Station TX).

## Results

Figure 2 shows the total average weekly units of high- and lower-sugar beverages sold per sequence for the six sequences according to message condition. The average percentage of high-sugar beverages sold was 54% in the control, 53% in the tax, and 54% in the subsidy message condition over the 9-week intervention period.

The average weekly units of high-sugar beverages sold during the control message condition was 115 beverages per vending machine. There was no difference in high-sugar beverages sold between the control and different message conditions as shown in Table 2. The weekly units of high-sugar beverages sold was on average 2 units lower for the tax message condition as compared with the control message condition (95% CI -8 to 5, *p* = 0.61). Further, during the subsidy message condition, it did not differ from the control message condition (95% CI -10 to 10, *p* = 0.96).

The average weekly units of lower-sugar beverages sold during control message condition was 98 beverages per vending machine. Table 2 shows that there was no difference in lower-sugar beverages sold between different message conditions. The weekly units of lower-sugar beverages sold was on average 4 units higher for the tax message condition as compared with the control message condition (95% CI -4 to 13, *p* = 0.32). Similarly, the weekly units of lower-sugar beverages sold was 7 units higher for the subsidy message condition as compared with the control message condition (95% CI -4 to 18, *p* = 0.18). Sensitivity analyses with adjustment for possible carryover effects did not change the conclusion.

**Table 2:** Effect of framed messages on high- and lower-sugar beverage sales using linear mixed effects models

Intervention	Average weekly units sold per machine (SD^a^)	Mean difference^c^ (95% CI^b^)	*P*-value
High-sugar beverages			
Control	115 (53)	0 (reference)	
Tax message	112 (57)	-2 (−8 to 5)	0.61
Subsidy message	117 (55)	0 (−10 to 10)	0.96
Lower-sugar beverages			
Control	98 (63)	0 (reference)	
Tax message	101 (65)	4 (−4 to 13)	0.32
Subsidy message	108 (71)	7 (−4 to 18)	0.18

^a^Denotes standard deviations

^b^Denotes confidence intervals

^c^Models adjusted for intervention period

## Discussion

Taxing SSB has been a topic of interest in recent years and the manner in which it is presented to the public is thought to be important in influencing consumer behaviour [38]. In our randomized cross-over trial, we evaluated the effect of framing an approximate 10% price difference between high- and lower-sugar beverages as ‘tax’ or ‘subsidy’ through placement of messaging materials on the beverage vending machines. Neither the tax message nor subsidy message substantially changed unit sales of high- or lower-sugar beverages as compared to the control condition with no messages in our study.

To our knowledge, there have been no previous studies that examined the effect of tax or subsidy framed messages alone on SSB purchases. Our finding that a subsidy message did not significantly impact the sales of high-sugar beverages is consistent with the result of a prospective interrupted time-series quasi-experiment [55]. The study reported that a 10% price discount for zero-calorie beverages increased the sales of zero-calorie beverages by 9.6% and led to a non-significant 2.2% increase in sales of SSB at cafeterias and convenience stalls located in two urban and one suburban hospitals in the U.S.. The intervention arm that included messaging in addition to the 10% discount (‘Lighten up for less – 10% off all zero-calorie bottled beverages and water’) through marketing posters, flyers and signs increased sales of zero-calorie beverages by 4.5% with a non-significant 1.4% decrease in SSB sales. These results and our findings suggest that the impact of tax and subsidy messages on sugary beverage purchase might be modest at best.

There are several potential reasons for the lack of effect of tax and subsidy messages on SSB sales in our study. First, the impact of a framing message depends on the degree it resonates with the audience [57]. The messages in our study were not crafted to target the beliefs or values of a specific group of individuals, which has been suggested to be more effective in promoting behavioural change [26, 58, 59]. Averseness to taxation could limit the impact of our messages due to feelings of impeachment of food choices freedom [36, 60]. Messages have also been found to be rejected by audiences when the source is not deemed to be credible [61]. The source of the messages was not stated in the intervention. If consumers deemed the source to be the vending machine vendor, then credibility of the information will be low as it might be seen as an advertisement with a vested interest to promote the healthier beverage sales as compared to a source that is perceived to be more reliable like the health authorities [62, 63].

Second, consumer’s choice of beverage can be strongly driven by their desire to satisfy their craving for certain beverages. In a qualitative study in U.S. college students, taste was mentioned as the most important factor for beverages choice with lower priced options only being chosen by those with lower allowances if taste of the beverage is not compromised [64]. Fixation to some SSB was mentioned to be akin to addiction by the American students overwriting knowledge of negative health impacts from consuming the beverages [64]. Similarly, in a qualitative study conducted in Mexican adolescents, most participants perceived a ~ 10% SSB tax to be ineffective in reducing their families’ or their own SSB intake due to the tax being too small to overcome SSB ‘addiction’ and taste preference [65]. The participants felt that the existing tax would only affect lower socio-economic status individuals’ SSB intake, and their own intakes will only be affected if the tax was higher. Tax and subsidy has been postulated to affect food/beverages consumption through the price elasticity of demand (percentage change in quantity demanded due to a percentage change in price, ceteris paribus) [41]. When consumers are “addicted”, the price elasticity of the beverage will tend to be lower i.e. change in beverage prices are less likely to have a pronounced effect on the consumption demand [66]. Also, consumers tend to be less sensitive to price of inexpensive goods as compared to expensive ones [66] and it is likely that the amount paid for beverages did not constitute a large proportion of young adults’ overall expenditures.

Third, consumption decisions may have been influenced by beverage companies’ strategic marketing messages targeted at consumers’ psychogenic needs (e.g. peer acceptance, pleasure and excitement) [7, 67–71]. Greater exposure to previous frames was suggested to weaken strength and lead to rejection of alternative frames that individuals are exposed to subsequently [72]. Loyalty to certain beverage brands could have influenced beverage choices in our study as well [73], we included a number of brands which were not offered previously and swapped certain brands (that were more popular) for another brand to ensure that both lower-sugar and high-sugar options were of the same brand.

A strength of our study was the real-world setting and the randomized cross-over design. This design improves comparability of the intervention groups, by minimising variance caused by factors such as location and presence of other competitor machines, as the vending machines served as their own control. The likelihood of bias due to changes in other determinants of sales was reduced as the machines were randomised into six intervention sequences. However, the statistical power might be limited by the low number of vending machines.

A further limitation of our study is that we did not include wash-out periods. This was due to the pragmatic nature of the study, to avoid encroaching into the vacation period when the overall sales is anticipated to be lower and hence contributing to a greater period effect. Carryover effects from message interventions during subsequent control periods could have caused the difference in beverage sales between message and control conditions to be smaller. However, our data analyses did not suggest that carryover effect was a cause of concern. Some level of contamination might have resulted from students in the university moving around the campus and being exposed to vending machines of the same company with a different message; the control condition was conducted simultaneously on the same campus. This might potentially confuse this group of consumers and reduce the credibility and persuasiveness of the message displayed as the consumers might have noticed that there was no price difference across these machines. Diet or weight changes were not measured in the population who purchased beverages from the machines, but substantial changes in diet or weight are unlikely to occur in the absence of significant changes in unhealthier beverage purchases in this study. Besides, the consumers of this study were mainly young adults from a university in Singapore and hence might not be representative of all adults in Singapore or in other countries. Compared to older adults, young adults might have lower prioritization of healthy eating and drinking habits due to low perceived risk of detrimental health conditions and hence are less easy to persuade to switch to lower-sugar options [64]. Our messages might be more salient and effective on adults with lower socio-economic status who are more sensitive to price differences [66].

Our findings suggest that the effect of adding subsidy and tax messages to price differences may have little impact on SSB purchases in vending machines. We are not certain if the effect of subsidy and tax messages will be similar in stores, supermarkets or other settings. Future studies should consider using mixed-methods research combining sales data collection with a qualitative study exploring key target groups’ perceptions and behavioural intent towards SSB consumption to help inform policy measures targeting SSB consumption reduction.

## Additional files

Additional file 1: **Table S1.** Characteristics of the machines in the six sequences. (DOCX 12 kb)

Additional file 2: **Table S2.** High-sugar beverages included in the study and their sugar content. **Table S3.** Lower-sugar beverages included in the study and their sugar content. (DOCX 14 kb)

Additional file 3: **Figure S1.** Images of the machines during ‘control’, ‘tax’ and ‘subsidy’ message conditions. (DOCX 1128 kb)

## Abbreviations

ANOVA: Analysis of variance
SSB: Sugar-sweetened beverages
