# Supplementary material for: Impact of tax and subsidy framed messages on high- and lower-sugar beverages sold in vending machines: a randomized crossover trial
Source: Int J Behav Nutr Phys Act. 2018 Aug 13;15:76. doi: 10.1186/s12966-018-0711-3 (PMC6090625; doi:10.1186/s12966-018-0711-3)
Supplement: Supplementary file 1 — Table S1. Characteristics of the machines in the six sequences. (DOCX 12 kb) [file 12966_2018_711_MOESM1_ESM.docx]

Supplementary Table 1: Characteristics of the machines in the six sequences

| **Characteristics** | **Sequence** | | | | | |
| --- | --- | --- | --- | --- | --- | --- |
|  | **1 (n=3)** | **2 (n=4)** | **3 (n=4)** | **4 (n=5)** | **5 (n=3)** | **6 (n=2)** |
| Number of machines with 30-slots selection panel | 3 | 4 | 1 | 5 | 3 | 2 |
| Number of machines with 36-slots selection panel | 0 | 0 | 3 | 0 | 0 | 0 |
| Number of machines located at sports facilities | 1 | 1 | 1 | 0 | 0 | 1 |
| Number of machines near other beverage competitor machine(s) | 3 | 4 | 4 | 4 | 2 | 2 |
| Number of machines near drinking water dispensers | 3 | 3 | 4 | 4 | 1 | 2 |
| Number of machines located at the canteen | 1 | 0 | 0 | 1 | 0 | 0 |
