# Supplementary material for: Impact of tax and subsidy framed messages on high- and lower-sugar beverages sold in vending machines: a randomized crossover trial
Source: Int J Behav Nutr Phys Act. 2018 Aug 13;15:76. doi: 10.1186/s12966-018-0711-3 (PMC6090625; doi:10.1186/s12966-018-0711-3)
Supplement: Supplementary file 2 — Table S2. High-sugar beverages included in the study and their sugar content. Table S3. Lower-sugar beverages included in the study and their sugar content. (DOCX 14 kb) [file 12966_2018_711_MOESM2_ESM.docx]

Supplementary Table 2: High-sugar beverages included in the study and their sugar content

| **No.** | **High-sugar Beverages** | **Price (SGD$)** | **Volume (ml)** | **Sugar content**  **per unit** | **Sugar content**  **per 100ml** | **Total calories (kcal) per unit** |
| --- | --- | --- | --- | --- | --- | --- |
| 1 | Heaven and Earth Jasmine Green Tea | 1.00 | 315 | 17.3 | 5.5 | 69 |
| 2 | Coke Regular | 1.00 | 330 | 35 | 10.6 | 140 |
| 3 | H2o Isotonic Sparkling | 1.10 | 500 | 43.5 | 8.7 | 175 |
| 4 | H2o Blackcurrant Sparkling | 1.10 | 500 | 35 | 7 | 140 |
| 5 | Sangaria Melon | 1.60 | 500 | 55 | 11 | 220 |
| 6 | Sangaria Grape | 1.60 | 500 | 61 | 12.2 | 245 |
| 7 | Sangaria Soda | 1.60 | 500 | 48.5 | 9.7 | 195 |
| 8 | 100 plus | 1.10 | 300 | 22.1 | 6.8 | 88 |
| 9 | Seasons' Ice Lemon Tea | 1.00 | 300 | 30 | 10 | 120 |
| 10 | Redbull | 1.10 | 250 | 40 | 16 | 167 |
| 11 | Milo | 1.00 | 240 | 16.1 | 6.7 | 144 |
| 12 | Bon café low fat Mocha | 1.00 | 240 | 18.7 | 7.8 | 126 |
| 13 | Bon café low fat Caffé Latte | 1.00 | 240 | 18.5 | 7.7 | 117 |

Supplementary Table 3: Lower-sugar beverages included in the study and their sugar content

| **No.** | **Lower Sugar Drinks** | **Price (SGD$)** | **Volume (ml)** | **Sugar content**  **per unit** | **Sugar content per 100ml** | **Total calories (kcal) per unit** |
| --- | --- | --- | --- | --- | --- | --- |
| 1 | Heaven and Earth Ayataka Green Tea | 0.90 | 315 | 0 | 0 | 0 |
| 2 | Coke Zero | 0.90 | 330 | 0 | 0 | 0 |
| 3 | Coke Light | 0.90 | 330 | 0 | 0 | 0 |
| 4 | Purified water | 0.70 | 500 | 0 | 0 | 0 |
| 5 | Jia Jia Liang Cha (No Sugar) | 0.90 | 300 | 0 | 0 | 0 |
| 6 | Sprite Zero | 0.90 | 300 | 0 | 0 | 0 |
| 7 | Redbull Low Sugar | 1.00 | 250 | 30.7 | 12.3 | 125 |
| 8 | Nescafe Mocha | 0.90 | 240 | 11.3 | 4.7 | 99 |
| 9 | Nescafe Latte | 0.90 | 240 | 10.8 | 4.5 | 99 |
