# Supplementary material for: Impact of tax and subsidy framed messages on high- and lower-sugar beverages sold in vending machines: a randomized crossover trial
Source: Int J Behav Nutr Phys Act. 2018 Aug 13;15:76. doi: 10.1186/s12966-018-0711-3 (PMC6090625; doi:10.1186/s12966-018-0711-3)
Supplement: Supplementary file 3 — Figure S1. Images of the machines during ‘control’, ‘tax’ and ‘subsidy’ message conditions. (DOCX 1128 kb) [file 12966_2018_711_MOESM3_ESM.docx]

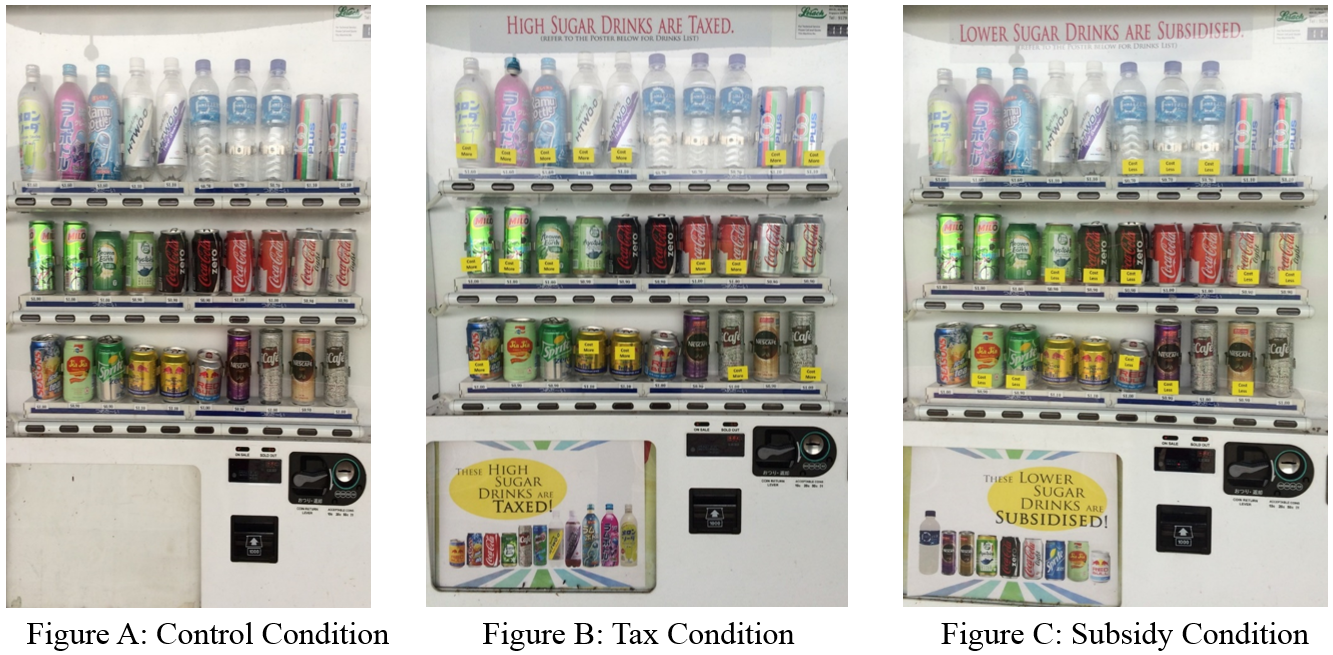


Supplementary Figure 1: Images of the machines during ‘control’, ‘tax’ and ‘subsidy’ message conditions
